# Pneumothorax, pneumomediastinum, pneumoperitoneum, and SCE after colonic ESD: a case report

**DOI:** 10.3389/fmed.2026.1885811

**Published:** 2026-10-01

**Authors:** Jianyi Zhang, Haoming Huang, Weichi Jiang, Xiliang Huang, Chunhua Fan, Yujin Wu

**Affiliations:** 1 Gastroenterology Department, The Affiliated Traditional Chinese Medicine Hospital, Guangzhou Medical University, Guangzhou, China; 2 Oncology Department, Guangdong Provincial Second Hospital of Traditional Chinese Medicine, Guangzhou, China

**Keywords:** endoscopic submucosal dissection, laterally spreading tumor, microperforation, pneumoperitoneum, pneumothorax, subcutaneous emphysema

## Abstract

**Background:**

Endoscopic submucosal dissection (ESD) has become a widely accepted technique for en bloc resection of colorectal laterally spreading tumors (LSTs). Although the procedure is generally considered safe, complications including bleeding and perforation may occur. The simultaneous development of pneumothorax, pneumomediastinum, pneumoperitoneum, and extensive subcutaneous emphysema following colorectal ESD represents an exceptionally rare but potentially serious adverse event.

**Case summary:**

A 56-year-old female patient underwent ESD for a granular-type LST measuring 8 mm × 20 mm located in the ascending colon. The procedure was performed under general anesthesia with tracheal intubation and carbon dioxide insufflation. Complete en bloc resection was achieved, and the post-ESD mucosal defect was closed with 16 titanium clips. Approximately 3 h after the procedure, the patient developed progressive neck swelling without respiratory distress or chest pain. Computed tomography (CT) revealed a small amount of pneumothorax on the right side and fluid pneumothorax on the left side (lung compression <10%), extensive subcutaneous emphysema involving the neck, chest wall, and right proximal thigh, pneumomediastinum, and pneumoperitoneum. The patient was managed conservatively with high-flow nasal cannula oxygen, bowel rest, intravenous antibiotics, and proton pump inhibitor therapy. Serial imaging demonstrated gradual resolution of the ectopic gas collections. The patient recovered uneventfully and was discharged on postoperative day 7.

**Conclusion:**

This case demonstrates that extensive gas extravasation involving multiple body compartments can occur as a rare complication of colorectal ESD, particularly when procedures are performed in the retroperitoneal portion of the colon under general anesthesia with positive pressure ventilation. Prompt recognition, timely CT evaluation, and conservative management in hemodynamically stable patients can lead to favorable outcomes without the need for surgical intervention.

## 1 Introduction

Colorectal cancer remains a major contributor to global cancer-related morbidity and mortality, and endoscopic removal of precursor lesions constitutes a fundamental strategy for cancer prevention (1). Laterally spreading tumors (LSTs) represent a distinct category of superficial colorectal neoplasms characterized by predominantly horizontal growth along the colonic wall, with variable malignant potential depending on their morphological subtype (2). Endoscopic submucosal dissection (ESD) has gained widespread acceptance as the preferred treatment modality for large LSTs, offering the advantages of en bloc resection, precise histopathological evaluation of resection margins, and low local recurrence rates (3).

Despite its established efficacy, ESD is technically challenging. The most commonly encountered complications include intraprocedural bleeding and perforation, with reported perforation rates ranging from 2% to 10% depending on lesion characteristics, anatomical location, and operator expertise (4). In rare instances, even clinically occult microperforations can lead to extensive gas extravasation along anatomical fascial planes, resulting in the simultaneous development of pneumothorax, pneumomediastinum, pneumoperitoneum, and widespread subcutaneous emphysema (5, 6).

These gas dissemination syndromes present unique diagnostic and management challenges. The clinical manifestations may be delayed, and the anatomical pathways of gas migration are complex, involving continuity between the retroperitoneum, mediastinum, pleural spaces, and subcutaneous tissues (7). Although isolated cases of such complications following gastrointestinal endoscopy have been documented (8, 9), the concurrent occurrence of pneumothorax, pneumomediastinum, pneumoperitoneum, and diffuse subcutaneous emphysema after colorectal ESD remains extremely uncommon (8, 10).

In this report, we describe a case of a patient who developed this constellation of complications following an uneventful ESD of an LST in the ascending colon. We provide a detailed analysis of the multifactorial pathogenesis, emphasizing the contributory roles of microperforation, anatomical susceptibility, procedural factors, and general anesthesia with positive pressure ventilation. The diagnostic approach and successful conservative management are discussed in the context of previously published cases.

## 2 Case report

### 2.1 Patient presentation and preoperative assessment

A 56-year-old female with no significant medical history presented for a routine screening colonoscopy. The patient reported no gastrointestinal symptoms, had no family history of colorectal cancer, and was not taking any antiplatelet or anticoagulant medications. Written informed consent was obtained following a thorough discussion of the procedure, its potential risks, and alternative treatment options.

### 2.2 Endoscopic procedure

The colonoscopy was performed on March 19, 2026, under general anesthesia with tracheal intubation to ensure optimal procedural conditions and airway protection. Carbon dioxide was used for luminal insufflation throughout the procedure.

A granular-type laterally spreading tumor was identified in the ascending colon, located 62 cm from the anal verge. The lesion measured approximately 8 mm in height and 20 mm in lateral diameter (Figure 1A). Narrow-band imaging revealed a regular microvascular pattern without features suggestive of deep submucosal invasion. A separate 1.2 cm type IIa polyp was noted in the sigmoid colon, 20 cm from the anal verge.

**FIGURE 1:**
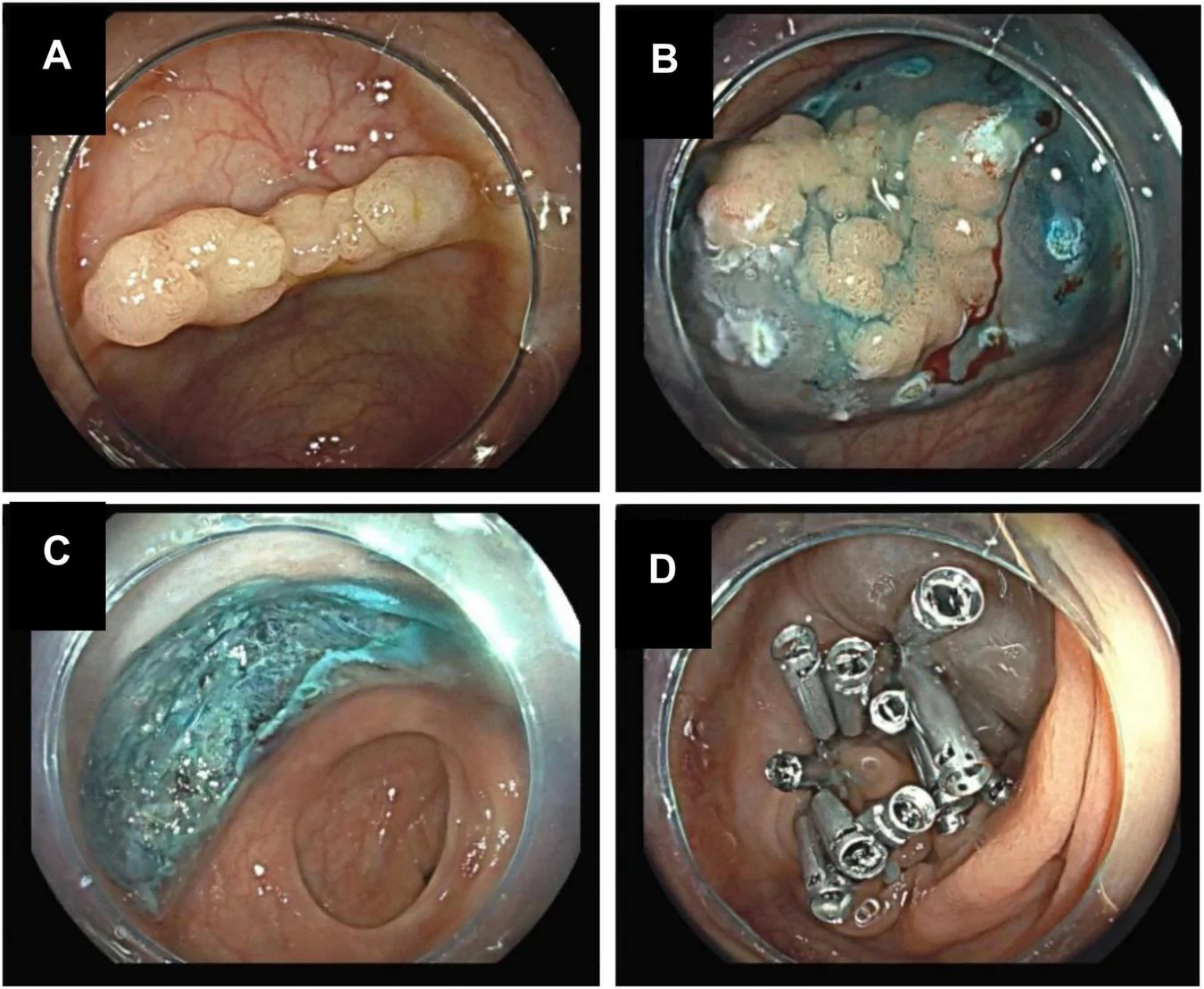
Endoscopic images of the ESD procedure. **(A)** Granular-type laterally spreading tumor in the ascending colon, measuring 8 mm × 20 mm. **(B,C)** Submucosal injection of glycerol-fructose and methylene blue solsolution. (Post-ESD mucosal defect after en bloc resection. **(D)** Closure of the mucosal defect with 16 titanium clips.

The ESD procedure for the ascending colon lesion was performed as follows:

#### 2.2.1 Marginal marking

Circumferential marking dots were placed 2–3 mm outside the visible tumor border using a disposable mucosal knife.

#### 2.2.2 Submucosal injection

A solution containing glycerol-fructose (Glyceol) and methylene blue was injected into the submucosal layer to create a sustained lifting cushion.

#### 2.2.3 Mucosal incision and dissection

A circumferential mucosal incision was made along the outer aspect of the marking dots, followed by meticulous submucosal dissection using the same knife. The lesion was resected en bloc without any intraprocedural complications. No overt perforation or deep muscular injury was observed during or immediately after the dissection (Figures 1B,C) .

#### 2.2.4 Wound closure

The post-ESD mucosal defect was closed prophylactically with 16 titanium clips to reduce the risk of delayed bleeding and perforation (Figure 1D).

The sigmoid colon polyp was resected via endoscopic mucosal resection (EMR), and the resulting defect was closed with two titanium clips.

The total procedure time was approximately 30 min. The patient was extubated uneventfully and transferred to the recovery unit in stable condition.

#### 2.2.5 CO_2_ insufflator model and settings

The procedure was performed using a Fujifilm CO_2_ insufflator (compatible with the Fujifilm endoscopic system). According to the manufacturer’s specifications, the Fujifilm CO_2_ insufflator features an adjustable flow rate range and a pressure range of 1–30 mmHg. The device also provides a maximum gas flow of up to 50 L/min.

During this ESD procedure, the insufflator was set to automatic pressure-controlled mode with the following parameters:

(1) Target pressure: Approximately 15–20 mmHg (within the device’s standard operating range)
(2) Flow rate: Automatically regulated by the device based on real-time intraluminal pressure and leakage; the flow rate typically varies between approximately 1–4 L/min under automatic pressure control

The automatic pressure-controlled mode is designed to maintain a stable intraluminal pressure by dynamically adjusting the flow rate according to detected pressure changes and gas leakage, thereby minimizing the risk of excessive insufflation.

#### 2.2.6 Estimated total volume of insufflated CO_2_

The ESD procedure lasted approximately 30 min. Based on the insufflator’s operational parameters in automatic pressure-controlled mode (with an average flow rate of approximately 1.4–2.0 L/min under stable conditions, as is commonly reported in ESD studies using CO_2_ insufflation), the estimated total volume of CO_2_ delivered during the procedure would be approximately:

- Low estimate: 1.4 L/min × 30 min = 42 L
- High estimate: 2.0 L/min × 30 min = 60 L

Thus, the total volume of CO_2_ insufflated during the 30-min procedure is estimated to be in the range of approximately 40–60 L. It should be noted that this represents the total gas output from the device; a portion of this gas would have been continuously lost through the natural gastrointestinal tract (via absorption, passive escape through the anus, or intermittent suction during the procedure), so the actual volume of gas retained intraluminally at any given time would be substantially lower.

#### 2.2.7 Intraoperative observation of luminal distension

Importantly, no excessive luminal distension was observed by the endoscopist during the entire procedure. The colonic lumen was maintained at a level sufficient for adequate visualization and meticulous submucosal dissection, without obvious over-distension or any signs of patient discomfort (the patient was under general anesthesia with tracheal intubation). The endoscopist periodically monitored the degree of luminal distension visually and adjusted insufflation as needed to maintain an optimal working field while avoiding over-insufflation.

### 2.3 Pathological findings

Histopathological examination of the ESD specimen from the ascending colon revealed a tubular adenoma. Both lateral and deep resection margins were free of neoplastic epithelium, confirming R0 resection. No submucosal invasion, high-grade dysplasia, or lymphovascular invasion was identified. The EMR specimen from the sigmoid colon was also confirmed to be a tubular adenoma with negative margins.

### 2.4 Postoperative course and complication onset

In the immediate postoperative period, the patient remained hemodynamically stable with a little discomfort in the throat, no abdominal pain, distension, or respiratory symptoms. She was instructed to fast and receive antibiotics after the operation.

Approximately 3 h after the procedure, the patient developed progressive neck swelling (Figure 2D). She denied chest pain, dyspnea, tachypnea, or abdominal discomfort. Physical examination revealed soft tissue swelling in the bilateral cervical region with palpable crepitus, consistent with subcutaneous emphysema. Vital signs remained stable: heart rate 88 beats/min, blood pressure 126/72 mmHg, respiratory rate 16 breaths/min, and oxygen saturation 98% on room air. Abdominal examination showed mild distension without tenderness, guarding, or rebound tenderness. Chest auscultation revealed clear breath sounds bilaterally.

**FIGURE 2:**
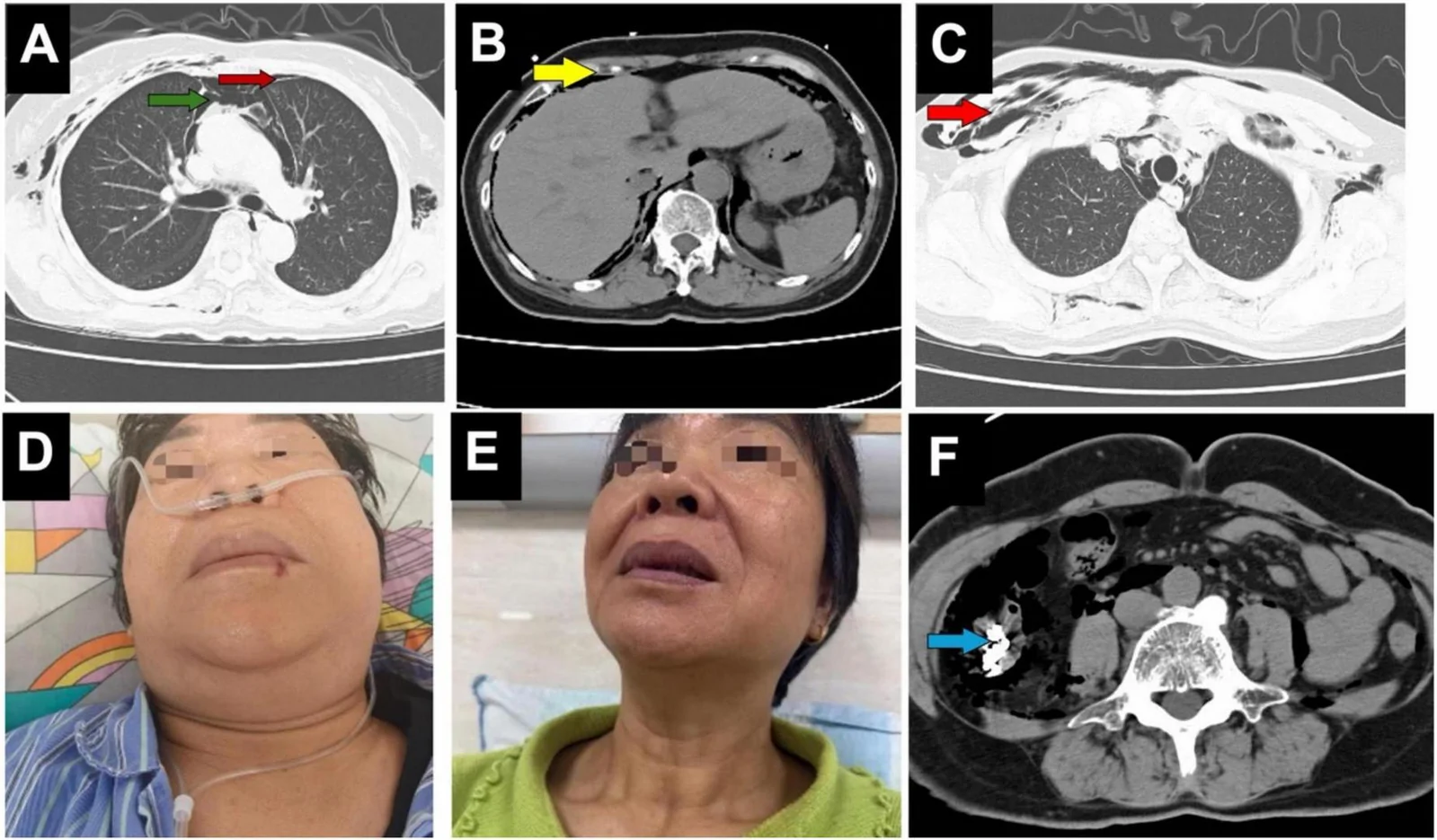
Computed tomography findings on postoperative day 1. **(A)** Axial CT (lung window) showing small left-sided pneumothorax (red arrow) and pneumomediastinum (green arrow). **(B)** Axial CT (abdominal window) demonstrating pneumoperitoneum (yellow arrow) and pneumoretroperitoneum involving the retroperitoneum, the mediastinum and lower chest wall, and the root of the right thigh. **(C)** Axial CT showing extensive subcutaneous emphysema in the neck (red arrows) and tracking into the right thigh root. **(D)** Before subcutaneous emphysema treatment. **(E)** After subcutaneous emphysema treatment. **(F)** The titanium clips placed at the ascending colon ESD site are clearly indicated by blue arrows.

Given the concern for possible gas extravasation, computed tomography (CT) scan was obtained on postoperative day 1.

### 2.5 Imaging findings

Contrast-enhanced CT of the neck, chest, abdomen, and pelvis revealed the following findings:

#### 2.5.1 Thoracic findings

There was a small amount of pneumothorax on the right side and hydropneumothorax on the left side. The lung tissue was compressed by less than 10% and there was a small amount of fluid (Figure 2A).

#### 2.5.2 Abdominal findings

Pneumoperitoneum symptoms: Accumulation of gas in the retroperitoneum, mediastinum, lower chest wall, and the root of the right thigh (Figure 2B).

#### 2.5.3 Subcutaneous emphysema

Multiple free gas shadows occur in the upper abdominal cavity and retroperitoneum, and there was extensive gas accumulation in the mediastinum, bilateral neck, bilateral temporal regions, left orbit, bilateral armpits and subcutaneous soft tissues of the chest and back (Figure 2C).

#### 2.5.4 Endoscopic clips

Metallic clips were identified in the proximal ascending colon and mid-to-distal sigmoid colon, corresponding to the ESD and EMR sites. No intra-abdominal fluid collections or abscesses were observed (Figure 2F).

### 2.6 Management and clinical course

A multidisciplinary team comprising gastroenterologists, Proctology experts, anesthesiologists, and radiologists was convened to guide management decisions. Given the absence of peritonitis, hemodynamic instability, and respiratory compromise, a conservative approach was adopted:

#### 2.6.1 Respiratory support

High-flow oxygen inhalation.

#### 2.6.2 Gastrointestinal management

The patient was instructed to fast for 72 h. Intravenous ilaprazole and broad-spectrum antibiotics (cefotaxime) were administered.

#### 2.6.3 Monitoring

Serial physical examinations, vital signs, and laboratory parameters, including white blood cell count and C-reactive protein, were closely monitored. The patient remained afebrile throughout with no elevation in inflammatory markers.

Over the subsequent 48 h, the neck swelling progressively subsided. On postoperative day 4, repeat CT imaging demonstrated marked reduction in the pneumothorax, pneumomediastinum, and subcutaneous emphysema. The patient was started on a clear liquid diet, which was well tolerated.

On postoperative day 5, the patient was transitioned to a low-residue diet. Antibiotics were discontinued after a 7-day course with no signs of infection.

### 2.7 Outcome and follow-up

The patient was discharged on postoperative day 7 in good condition with complete resolution of all gas-related findings (Figure 2E). She was advised to avoid strenuous activities for 1 week and to undergo surveillance colonoscopy at 6 months. At a telephone follow-up, the patient reported no symptoms and had returned to her normal activities.

## 3 Discussion

This case illustrates a rare but significant complication of colorectal ESD: the development of pneumothorax, pneumomediastinum, pneumoperitoneum, and extensive subcutaneous emphysema following an otherwise uneventful procedure. The occurrence of such widespread gas extravasation is rarely attributable to a single factor; rather, it likely results from the convergence of multiple predisposing elements. In this discussion, we provide a detailed multifactorial analysis of the potential causes, including the roles of microperforation, thermal injury, anatomical susceptibility, procedural factors, and, importantly, the impact of general anesthesia with tracheal intubation and positive pressure ventilation.

### 3.1 The central role of microperforation

No overt colonic perforation was visualized during or immediately after the ESD procedure. The post-ESD defect was closed with clips. The patient felt discomfort in the throat after the operation. This patient had no abdominal pain or rebound tenderness, and no peritoneal irritation signs. The discomfort in the throat after the operation, coupled with the absence of peritonitis or hemodynamic instability, supports that there are other causes other than microperforation that have led to this complication.

Microperforation refers to a transmural defect that is too small to be detected endoscopically but permits the egress of intraluminal gas into extraluminal spaces (11). In a series of 823 gastric ESD procedures, microperforations occurred in 2.1% of cases and were associated with longer procedure times and lesions located in the upper stomach (12). For colorectal ESD, microperforations are likely underreported, as they often remain clinically silent or manifest only with subtle, delayed symptoms.

### 3.2 Anatomical susceptibility: the retroperitoneal location

The location of the lesion in the ascending colon is critically important. The ascending colon is retroperitoneal in its posterior aspect, and lesions involving this segment may predispose to retroperitoneal rather than intraperitoneal gas dissemination (13). In our patient, the presence of pneumoretroperitoneum on CT supports retroperitoneal origin of the gas leak.

From the retroperitoneum, gas can dissect along several anatomical pathways:

#### 3.2.1 Cephalad extension

Gas may travel along retroperitoneal fascial planes, including the perirenal and retrocrural spaces, into the mediastinum, causing pneumomediastinum (14).

#### 3.2.2 Pleural involvement

Mediastinal gas may rupture into the pleural cavity, resulting in pneumothorax (5).

#### 3.2.3 Anterior extension

Gas may pass through the posterior parietal peritoneum into the peritoneal cavity, causing pneumoperitoneum (8).

#### 3.2.4 Peripheral extension

Gas may dissect laterally and inferiorly along fascial planes of the abdominal wall, flank, and pelvic regions, extending to the thigh roots, as observed in this case (6).

This pattern of gas migration – from retroperitoneal microperforation to mediastinum, pleura, peritoneum, and subcutaneous tissues – has been well documented in the literature (7, 9, 15).

### 3.3 Procedural risk factors for microperforation

Several factors related to the ESD procedure itself may have contributed to the development of microperforation (16):

#### 3.3.1 Thermal injury

The use of electrocautery during submucosal dissection can cause thermal injury to the muscularis propria, even when the dissection plane is maintained in the submucosa. This may result in delayed transmural necrosis and microperforation hours after the procedure (17).

#### 3.3.2 Prolonged procedure duration

Prolonged ESD has been identified as a risk factor for perforation, likely due to cumulative thermal injury and repeated manipulation of the submucosal plane (18). The total procedure time was 30 min.

#### 3.3.3 Submucosal injection technique

Although submucosal injection is intended to create a safety cushion, needle insertion itself may inadvertently create micro-injuries in the intestinal wall, particularly in areas where the submucosa is thin (19). In the ascending colon, the submucosal layer can be tenuous, increasing this risk.

#### 3.3.4 Insufflation gas

Carbon dioxide (CO_2_) was used for insufflation. While CO_2_ is preferred over air due to its rapid absorption and reduced post-procedural discomfort, it does not eliminate the risk of gas extravasation (20). Once a microperforation occurs, continuous insufflation of any gas can act as a driving force, pushing gas into the retroperitoneal space. However, the rapid absorption of CO_2_ may paradoxically accelerate the resolution of gas collections once the leak is sealed, as observed in our patient.

### 3.4 The contribution of general anesthesia with positive pressure ventilation

A distinctive feature of this case is the use of general anesthesia with tracheal intubation and positive pressure ventilation (PPV). While this approach ensures a stable airway and optimal procedural conditions for complex ESD, it introduces additional risk factors that may synergize with a colonic microperforation to produce widespread gas extravasation.

#### 3.4.1 Increased intra-thoracic and intra-abdominal pressure

PPV generates positive pressure throughout the thoracic cavity, which is transmitted to the abdominal cavity via the diaphragm, resulting in elevated intra-abdominal pressure. This increased pressure can act as a driving force, exacerbating the egress of intraluminal gas through a microperforation and promoting its rapid dissection along fascial planes (21, 22).

#### 3.4.2 Barotrauma as a potential contributing factor

Although less likely as a primary cause given the imaging findings, PPV itself carries a risk of barotrauma, which can lead to pneumothorax, pneumomediastinum, and subcutaneous emphysema (10). This can occur due to alveolar rupture from high airway pressures, particularly if there is underlying lung pathology or if ventilation parameters are not carefully optimized. In this case, the initial CT finding of a small pneumothorax could theoretically have two origins: (1) extension of retroperitoneal gas into the pleural space (most likely, given the continuity of gas along mediastinal planes), or (2) primary barotrauma from PPV (less likely, as the gas pattern was contiguous with the retroperitoneal and mediastinal gas). Nevertheless, it is plausible that PPV contributed to the extent of the pneumothorax once mediastinal gas was present.

#### 3.4.3 Delayed recognition

Under general anesthesia, the early clinical signs of an evolving complication – such as patient agitation, tachypnea, or abdominal distension – may be masked or attributed to anesthesia-related factors. This can delay recognition and allow the process to progress. In our patient, symptoms only became apparent 3 h post-extubation, after the patient had awakened and was able to report neck swelling.

The interaction between PPV and a retroperitoneal microperforation creates a particularly high-risk scenario. The PPV elevates intra-abdominal pressure, forcing gas out through the defect. The retroperitoneal location allows that gas to travel unimpeded along low-resistance fascial planes, while the ongoing PPV provides the sustained pressure gradient to drive gas to distant anatomical sites. This synergistic mechanism best explains the extensive and multi-compartmental nature of the gas extravasation observed in this case.

### 3.5 A multifactorial pathogenic model

Based on the analysis above, we propose a multifactorial model for the pathogenesis of this complication, which can be conceptualized as a sequence of events:

#### 3.5.1 Initiating event

A microperforation occurs in the retroperitoneal portion of the ascending colon, likely due to a combination of thermal injury from electrocautery and the inherent fragility of the colonic wall at that site.

#### 3.5.2 Driving force

Positive pressure ventilation during general anesthesia increases intra-abdominal pressure, creating a pressure gradient that forces gas from the colonic lumen into the retroperitoneal space.

#### 3.5.3 Anatomical conduit

The retroperitoneal space acts as a pathway of low resistance, allowing gas to dissect cephalad into the mediastinum, anteriorly into the peritoneum, and laterally/inferiorly into the chest wall, neck, and thighs.

#### 3.5.4 Secondary manifestations

Mediastinal gas ruptures into the pleural space, causing pneumothorax. Gas also extends into the peritoneal cavity (pneumoperitoneum) and along fascial planes (subcutaneous emphysema).

#### 3.5.5 Delayed presentation

The microperforation may seal spontaneously (aided by clip closure and the natural healing response), but the gas already extravasated persists. Symptoms only become apparent after the patient awakens from anesthesia and when the gas accumulation reaches a threshold that causes physical distension or when the patient can articulate discomfort.

This model highlights that the complication was not due to a single error, but rather the unfortunate convergence of multiple risk factors. It underscores the importance of a high index of suspicion, especially in patients undergoing prolonged ESD in the right colon under general anesthesia.

### 3.6 Comparison with previously reported cases

The pattern of gas extravasation in our patient is similar to that reported by Liu et al. in a 2025 case report (6). In their case, a 55-year-old female undergoing ESD for an ascending colon polyp developed bilateral massive pneumothorax, pneumomediastinum, pneumoperitoneum, and grade V subcutaneous emphysema. A key difference is that their patient developed acute respiratory distress intraoperatively and required immediate chest tube drainage. This more severe and acute presentation may be explained by the use of air insufflation (rather than CO_2_) and possibly a larger or more extensive microperforation. Notably, their patient was also under general anesthesia with PPV, further supporting the role of PPV as a critical co-factor in driving gas extravasation.

A smaller case report from Jang et al. in *Endoscopy* described left-sided pneumothorax and pneumoperitoneum following gastric ESD under conscious sedation, which resolved with conservative management including pigtail catheter drainage (23). The absence of PPV in that case may explain why the gas extravasation was less extensive than in our patient or the case by Liu et al. (6).

These comparisons suggest that the combination of retroperitoneal microperforation and positive pressure ventilation is a particularly high-risk scenario that can lead to dramatic and widespread gas dissemination.

### 3.7 Diagnostic and management considerations

The diagnosis of gas extravasation complications relies on a high index of suspicion and appropriate imaging. In our patient, the onset of neck swelling with crepitus prompted immediate CT evaluation.

CT is the imaging modality of choice for characterizing ectopic gas collections (24). It provides detailed information on:

1. The extent and distribution of gas (pneumothorax, pneumomediastinum, pneumoperitoneum, subcutaneous emphysema).
2. The presence of fluid collections or abscesses.
3. The potential site of perforation (by correlating with endoscopic clip locations).
4. Signs of tension physiology (mediastinal shift, tracheal deviation, cardiac compression).

Plain chest and abdominal radiographs may reveal free air under the diaphragm, pneumothorax, or subcutaneous gas, but they lack the sensitivity and anatomical detail of CT (25). In resource-limited settings, serial radiographs may be used to monitor disease progression, but CT remains the gold standard for initial diagnosis and treatment planning.

The management of gas extravasation following endoscopic perforation depends on several factors: the patient’s clinical stability, the extent of gas dissemination, the presence of peritonitis or sepsis, and the likelihood of ongoing gas leak (26). In our patient, conservative management was successful due to several favorable features:

1. Hemodynamic stability with no signs of shock

2. Absence of peritonitis

3. Small-volume pneumothorax without tension physiology

4. No intra-abdominal fluid or abscess on CT

5. Clip closure of the mucosal defect, likely sealing the microperforation

Conservative measures included high-flow oxygen inhalation (to promote gas reabsorption), bowel rest (to reduce intraluminal pressure), and prophylactic antibiotics (to prevent secondary infection of gas collections) (21, 27). Close monitoring with serial physical examinations, vital signs, and laboratory markers was essential to detect early signs of deterioration.

Surgical intervention is indicated in cases with hemodynamic instability, septic shock, tension pneumothorax, peritonitis, or failure of conservative management with progressive gas accumulation.

### 3.8 Preventive strategies for gas-related complications during colorectal ESD

The present case highlights the importance of implementing preventive strategies to minimize the risk of extensive gas extravasation during colorectal ESD. Although such complications are rare, the potentially serious consequences underscore the need for proactive measures, particularly when operating in the retroperitoneal colon under general anesthesia with positive pressure ventilation. Based on our experience and a review of the literature, several strategies may be considered.

First, the use of CO_2_ insufflation is strongly recommended by the European Society of Gastrointestinal Endoscopy (ESGE) Technical Review (28). CO_2_ is absorbed more rapidly than room air and is eliminated through the lungs, which reduces the risk of tension pneumoperitoneum and extensive subcutaneous emphysema in the event of perforation. However, as demonstrated in our patient, even with CO_2_ insufflation, extensive gas extravasation can occur in the presence of microperforation, highlighting the need for additional preventive measures beyond simply choosing the insufflation gas.

Second, minimizing intraluminal pressure is of paramount importance. Procedural strategies to achieve this include using the lowest effective insufflation pressure, avoiding excessive insufflation, and intermittently aspirating intraluminal gas during the procedure. The use of transparent caps may also reduce the amount of insufflated gas required by improving visualization and maintaining a stable working distance. In addition, maintaining the patient in an appropriate position (e.g., avoiding the Trendelenburg position when feasible) and minimizing the duration of positive pressure ventilation may help reduce the driving force for gas extravasation in patients with an unrecognized microperforation (29).

Third, emerging low-pressure ESD techniques offer promising alternatives that may theoretically reduce the risk of extensive gas extravasation. Underwater ESD (UESD) is performed with saline immersion, which reduces or eliminates the need for gas insufflation. A recent systematic review and meta-analysis by Singh et al. (30), including seven studies with 1,401 patients, found that UESD had both a shorter procedure time and greater dissection speed when compared with conventional ESD, with no significant differences in en-bloc resection, R0 resection, or adverse events including perforation. A randomized controlled trial by Oh et al. (31) comparing UESD and conventional ESD for large laterally spreading tumors (20–50 mm) demonstrated that UESD was superior in total procedure time and dissection speed, with no perforations observed in either group. UESD may be particularly beneficial for lesions where gas extravasation is a concern, as the water environment provides a physical barrier that limits gas escape in the event of microperforation. Other gas-free saline-immersion techniques, such as the saline-immersion/irrigation technique (SITE), have also emerged as alternative platforms that offer improved visualization, natural buoyancy providing intrinsic traction, heat-dissipation limiting collateral tissue damage, and reduced luminal distension (32). The water pressure method (WPM), which uses active water pressure via the waterjet function of an endoscope, has been shown to be a helpful technique for overcoming difficulties with colorectal ESD (33).

Gel immersion ESD (Gi-ESD) represents another innovative approach that offers benefits such as buoyancy, traction, and a clear visual field without gas insufflation. Nakano et al. (34) reported the successful use of gel immersion ESD using the tunneling method for a 50-mm laterally spreading tumor in the ascending colon, demonstrating that the gel provides excellent visualization even during bleeding, and its viscosity offers a better and long-lasting submucosal cushion (33). Clinical application of a dedicated gel with appropriate viscosity to prevent mixing with blood has been reported to be effective in securing the visual field during endoscopy (34). These features make Gi-ESD a promising alternative for reducing gas-related complications, particularly in cases where extensive gas extravasation is a concern (35).

Fourth, routine postoperative imaging may be considered in selected high-risk patients. Although not recommended for all patients, the detection of extraluminal gas by CT in a significant proportion of patients after colorectal ESD warrants attention. Kobayashi et al. (36) conducted a prospective study of 91 patients who underwent colorectal ESD and found that extraluminal gas was observed in 31 of 91 patients (34%) and was significantly correlated with the occurrence of post-ESD inflammatory syndrome (PEIS), even in cases without obvious intraoperative perforation or penetration. This finding underscores the importance of preventive strategies and careful postoperative monitoring, even when no overt perforation is observed during the procedure.

Finally, a high index of suspicion for delayed gas extravasation syndromes is essential, particularly after procedures involving the right colon under general anesthesia. Prompt recognition, early CT evaluation, and multidisciplinary collaboration are key to optimizing patient outcomes when such complications do occur. Future prospective studies are needed to compare the safety profiles of different preventive strategies and to establish evidence-based guidelines for minimizing gas-related complications during colorectal ESD.

## 4 Conclusion

We report a case of extensive gas extravasation – including pneumothorax, pneumomediastinum, pneumoperitoneum, and widespread subcutaneous emphysema – following ESD of a laterally spreading tumor in the ascending colon. This rare complication likely resulted from a retroperitoneal microperforation with subsequent gas dissection along fascial planes, exacerbated by positive pressure ventilation during general anesthesia.

This case underscores several important lessons:

1. Microperforation may occur despite an apparently uneventful ESD and can present with delayed symptoms.
2. The combination of retroperitoneal lesion location and general anesthesia with positive pressure ventilation creates a high-risk scenario for widespread gas dissemination.
3. CT is essential for accurate diagnosis and characterization of the extent of gas extravasation.
4. In hemodynamically stable patients without peritonitis or tension physiology, conservative management with high-flow oxygen, bowel rest, and antibiotics can achieve favorable outcomes without surgical intervention.

Clinicians performing colorectal ESD should maintain a high index of suspicion for delayed gas extravasation syndromes, particularly after procedures involving the right colon under general anesthesia. Prompt recognition, multidisciplinary collaboration, and appropriate conservative management are key to optimizing patient outcomes.

## Data Availability

The original contributions presented in the study are included in the article/supplementary material, further inquiries can be directed to the corresponding authors.
